# Molecular Evolutionary Analysis of pH1N1 2009 *Influenza* Virus in Reunion Island, South West Indian Ocean Region: A Cohort Study

**DOI:** 10.1371/journal.pone.0043742

**Published:** 2012-08-27

**Authors:** Hervé Pascalis, Sarah Temmam, David A. Wilkinson, Najla Dsouli, Magali Turpin, Xavier de Lamballerie, Koussay Dellagi

**Affiliations:** 1 GIS CRVOI, Centre de Recherche et de Veille sur les maladies émergentes dans l’Océan Indien, Plateforme technologique CYROI, Saint-Denis, La Réunion, France; 2 Institut de Recherche pour le Développement (IRD), La Réunion, France; 3 Ecologie microbienne (UMR 5557) CNRS-Université de Lyon, Lyon, France; 4 Université de La Réunion, Saint-Denis, La Réunion, France; 5 Unité des Virus Emergents (UMR-S 190), IRD-Université de la Méditerranée, Marseille, France; Faculty of Biochemistry Biophysics and Biotechnology, Jagiellonian University, Poland

## Abstract

**Background/Objectives:**

Molecular epidemiology is a powerful tool to decipher the dynamics of viral transmission, quasispecies temporal evolution and origins. Little is known about the pH1N1 molecular dynamics in general population. A prospective study (CoPanFlu-RUN) was carried out in Reunion Island to characterize pH1N1 genetic variability and molecular evolution occurring in population during the pH1N1 *Influenza* pandemic in 2009.

**Methodology:**

We directly amplified pH1N1 genomes from 28 different nasal swabs (26 individuals from 21 households). Fifteen strains were fully sequenced and 13 partially. This includes pairs of sequences from different members of 5 separate households; and two pairs from individuals, collected at different times. We assessed the molecular evolution of pH1N1 by genetic variability and phylogenetic analyses.

**Principal Findings:**

We found that i) Reunion pH1N1 sequences stemmed from global “clade 7” but shaped two phylogenetic sub-clades; ii) D239E mutation was identified in the hemagglutinin protein of all Reunion sequences, a mutation which has been associated elsewhere with mild-, upper-respiratory tract pH1N1 infecting strains; iii) Date estimates from molecular phylogenies predicted clade emergence some time before the first detection of pH1N1 by the epidemiological surveillance system; iv) Phylogenetic relatedness was observed between Reunion pH1N1 viruses and those from other countries in South-western Indian Ocean area; v) Quasispecies populations were observed within households and individuals of the cohort-study.

**Conclusions:**

Surveillance and/or prevention systems presently based on *Influenza* virus sequence variation should take into account that the majority of studies of pH1N1 *Influenza* generate genetic data for the HA/NA viral segments obtained from hospitalized-patients, which is potentially non-representative of the overall viral diversity within whole populations. Our observations highlight the importance of collecting unbiased data at the community level and conducting whole genome analysis to accurately understand viral dynamics.

## Introduction

The first *Influenza* pandemic of the 21^st^ century was caused by the 2009 A/H1N1 *Influenza* virus (pH1N1), first reported in early spring 2009 in Mexico and the United States [1]. Initial phylogenetic studies showed that this virus was a reassortant of genomic segments from an Eurasian lineage swine H1N1 *Influenza* virus and a North American triple-reassortant swine H1N2 or H1N1 virus [2], [3]. From July 19 the new virus spread across the world, reaching more than 140 countries [4]. The early viral diversification into seven discrete genetic clades [5] was further confirmed by several subsequent studies [6]–[9]. Clade 7 rapidly became the most prevalent worldwide, but other clade-variants continued to circulate, as most countries were affected by pH1N1 through multiple introductions of different clade members [6], [8], [10]–[14]. These multiple introductions are likely explained by the air-borne transmission of flu [15] and by intense international air traffic and exchanges [12]. International aircraft travel on which passengers typically are confined for a period of hours present opportunities for air borne transmission. Airborne viral diseases as in the case of *Influenza* are more prone during the preclinical incubation period to silent transmission and large diffusion among travellers of the same flight and hence are more likely associated with multiple undetected introductions in a given country. Once introduced, new viral strains are likely to spread rapidly across geographic regions [16].

The epidemic wave of the *Influenza* pandemic caused by pH1N1 reached Reunion Island during the austral winter 2009 (July–December). According to the department of epidemiological surveillance, the epidemic activity started on week 30 (July 20), peaked on week 35 (August 24) and was completely over by week 40 (September 28) [17]. The first case imported from Australia was detected on July 5 and the first autochthonous transmission was recorded on July 21. First estimates suggested that 67,000 individuals who had consulted a physician were infected by the pH1N1 virus [17]. However, these studies were largely skewed towards symptomatic patients seeking medical support and their conclusions could hardly be extrapolated to ILIs occurring in the community.

The CoPanFlu programme is an international project dedicated to the study of the pandemic, based on the follow-up of household cohorts in metropolitan France, Reunion Island [18] and other African [19], South-American and Asian countries. In Reunion Island, the CoPanFlu-RUN programme provided the first pH1N1 sero-epidemiological analyses and revealed that the number of individuals infected by pH1N1 was at least 3 times higher than the estimates based on epidemiologic surveillance data, mainly as a consequence of the mild or unapparent disease that escaped medical attention [18].

Here we report on the genetic variability and molecular evolution of pH1N1 viruses characterized during this prospective follow-up programme and attempt to understand their evolutionary implications in the geographic context of the South West Indian Ocean (SWIO) region.

## Materials and Methods

### Clinical Samples

The CoPanFlu-RUN prospective study was conducted between July 21 (week 30) and October 31 2009 (week 44) The CoPanFlu-RUN cohort was selected to be representative of the whole Reunion Island population. We took special attention to select households representing a wide range of geographic locations in order to minimize the repartition bias. For more details about the cohort design, see [18]. A total of 772 households (2,164 individuals) were included in the study. An active telephonic inquiry was conducted to record *Influenza*-Like Illness (ILI) symptoms occurring in households. Reports of ILI, defined as documented fever (≥37.8°C) with at least one symptom of Upper Respiratory Tract Infection (URTI: sore throat, cough, running and or stopped nose or systemic symptom like aching), in at least one member of a household, led to 3 consecutive visits by a nurse (at days 0, 3 and 8 post-report), during which nasal swabs were collected from all family members regardless of their clinical presentation. ILI alerts were managed for the study period and led to the collection of 1,196 nasal swabs belonging to 443 individuals living in 125 households.

### Sample Nomenclature

Reunion Island sequences were labelled according to the origin of the sample, whilst retaining anonymity of the participants : For example, “2133-5-M1E” corresponds to the fifth member of the family designated as number “2133”, whereas “2133-6-M1E” refers to the sixth household member. “M1E”, “M2E” and “M3E” refer to the first, second or third sampling performed at days 0, 3 or 8 post ILI report, respectively. For households that were sampled twice because of the recurrence of ILI alerts, pH1N1 viruses identified in the three successive sampling events were designated as “M4E” “M5E” or “M6E”.

### Detection of pH1N1

All nasal swab samples (Sigma-Virocult®, Medical Wire [MWE]) were spiked before nucleic acid extraction with an internal RNA phage control [20]. RNA was extracted from 140 µL of swab supernatant using the QIAamp Viral RNA Mini Kit (Qiagen), according to the manufacturer’s protocol.

Samples were subsequently screened for the presence of *Influenza* A virus RNA by qRT-PCR using a pan-*Influenza* A SYBR Green qRT-PCR assay targeting the M gene [21] (Quantitect SYBR Green qRT-PCR, Qiagen). pH1N1 detection was assessed using a pH1N1-specific TaqMan probe qRT-PCR assay targeting the HA gene (SuperScript III Platinum one-step qRT-PCR system, Invitrogen), according to the recommendations of the Pasteur Institute (Van der Werf, S. & Enouf, V., SOP/FluA/130509).

### Genome Sequencing and Alignment

Out of the 101 nasal swabs detected positive for pH1N1 (corresponding to 62 individuals) we decided to sequence 28 strains (corresponding to 26 individuals belonging to 21 households) distributed so as to reflect the epidemiologic and temporal dynamic of the epidemics in the cohort [18]: epidemic (W33–37, n = 26) and post-epidemic (W38–40, n = 2) periods. No nasal swabs were detected positive for pH1N1 during the pre-epidemic period (W30–32).

Reverse transcription was performed on freshly extracted RNA using the Superscript III Reverse Transcriptase (Invitrogen, USA), according to the manufacturer’s instructions, then cDNA was aliquoted and stored at −80°C. For genetic characterization of viruses, genome amplification (and subsequent sequencing) was performed from RNAs directly extracted from nasal swabs without resorting to cell-culture viral isolation, to avoid any significant alteration in the representation of the different viral populations present in each swab [22]. All PCR amplifications were performed with the Hot Start Taq DNA Polymerase (Promega, France) and a specific set of primers designed for this study (primers available on request), based on a consensus sequence of all the complete sequences of pH1N1 available at the day of design (2009). To sequence the complete open reading frames (ORFs) of each segment, primers were designed to amplify 3–6 overlapping fragments, providing a minimum of 3-fold genetic coverage. Briefly, amplifications were performed with 200 nM of each primer, 2.5 mM of MgCl_2_, 0.2 mM of dNTP, 1.25 U of Hot Start Taq DNA polymerase and 5 µL of cDNA. Cycling conditions were: 95°C - 2 min followed by 35 cycles of 94°C - 5 sec, Tm - 30 sec, 72°C - 1 min 30 and a final elongation step at 72°C - 7 min. When needed, nested-PCR reactions were performed to increase the amount of cDNA. In this particular cases, PCR reactions were performed as described above, except that only 20 cycles were ran for the first PCR round, following by 35 cycles for the second PCR round, in order to minimize mutations introduced by PCR reactions. Sequences alignments were trimmed and assembled in Geneious Pro 5.3.4 software package [23] using MUSCLE alignment method [24].

### Phylogenetic Analysis

pH1N1 viruses from the CoPanFlu-RUN cohort selected for sequencing were temporally distributed over the full epidemic wave 2009 (weeks 33–42). Concatemers of all 8 segments (“Concat-8”) from 15 fully sequenced viruses (ordered PB2, PB1, PA, HA, NP, NA, M and NS; complete ORFs) or of 6 segments (“Concat-6”) from 13 partially-sequenced viruses (ordered PA, HA, NP, NA, M and NS; complete ORFs) were generated for phylogenetic analysis. Concatemers were similarly generated for 101 GenBank sequences for phylogenetic comparison. GenBank sequences used for phylogenetic analyses were selected on their belonging to the 7 world clades [5], [6] and to their temporal distribution in 2009 (sequences ranging from April 01 to October 17, 2009). GenBank accession numbers of sequences from Reunion Island are from JQ431196-JQ431393 and the full details of the sequences are listed in Table 1.

**Table 1:** Epidemiological data for pH1N1 Reunion Island viruses from the CoPanFlu-RUN cohort.

Sequence name	Date of collection(day D0, D3, D8)	Age(years)	Sex	Householdmember	Same individualat D0 & D3	Symptoms*	GenBank acc. n°(ordered PB2 - PB1 - PA - HA - NP - NA - M - NS)
A/Reunion/0116-3-M1E/2009(H1N1)	2009/09/05 (D0)	12	M	no	I2	ILI	JQ431-387/375/358/200/291/257/228/308
A/Reunion/0116-3-M2E/2009(H1N1)	2009/09/07 (D3)					URTI	−/−/JQ431-359/201/292/258/225/309
A/Reunion/0148-4-M1E/2009(H1N1)	2009/08/19 (D0)	20	M	H3	no	ILI	JQ431-389/374/353/210/281/260/233/310
A/Reunion/0148-6-M1E/2009(H1N1)	2009/08/19 (D0)	13	F		no	ILI	JQ431-390/376/355/215/282/261/224/311
A/Reunion/0159-3-M1E/2009(H1N1)	2009/08/25 (D0)	8	M	no	no	ILI	JQ431-383/377/344/211/293/262/234/312
A/Reunion/0215-4-M1E/2009(H1N1)	2009/09/04 (D0)	13	M	no	I1	ILI	JQ431-380/370/342/198/294/263/226/313
A/Reunion/0215-4-M2E/2009(H1N1)	2009/09/07 (D3)					URTI	−/−/JQ431-339/199/295/264/227/314
A/Reunion/1572-3-M1E/2009(H1N1)	2009/08/21 (D0)	21	F	no	no	ILI	−/−/JQ431-356/216/283/265/235/315
A/Reunion/1632-5-M1E/2009(H1N1)	2009/08/18 (D0)	13	M	no	no	ILI	−/−/JQ431-337/217/296/266/236/316
A/Reunion/1658-4-M1E/2009(H1N1)	2009/08/14 (D0)	2	M	no	no	ILI	−/−/JQ431-361/212/297/267/237/317
A/Reunion/1698-3-M1E/2009(H1N1)	2009/09/28 (D0)	9	M	no	no	ILI	−/−/JQ431-354/202/298/259/238/318
A/Reunion/1709-4-M2E/2009(H1N1)	2009/08/22 (D3)	5	M	no	no	ILI	JQ431-384/378/360/218/299/268/239/319
A/Reunion/1722-7-M1E/2009(H1N1)	2009/08/13 (D0)	7	F	H4	no	ILI	−/−/JQ431-348/206/284/269/240/320
A/Reunion/1722-8-M1E/2009(H1N1)	2009/08/13 (D0)	4	M		no	ILI	−/−/JQ431-349/207/285/270/241/321
A/Reunion/2133-5-M1E/2009(H1N1)	2009/08/24 (D0)	23	M	H1	no	ILI	JQ431-381/369/345/208/286/271/242/322
A/Reunion/2133-6-M1E/2009(H1N1)	2009/08/24 (D0)	3	M		no	ILI	JQ431-391/368/350/209/287/272/243/323
A/Reunion/2154-2-M5E/2009(H1N1)	2009/09/05 (D3)	43	M	H2	no	URTI	JQ431-392/364/351/203/288/278/229/324
A/Reunion/2154-4-M4E/2009(H1N1)	2009/09/02 (D0)	15	F		no	ILI	JQ431-385/365/352/204/289/279/230/325
A/Reunion/2224-3-M3E/2009(H1N1)	2009/08/26 (D8)	11	M	no	no	neg	JQ431-393/371/362/219/300/252/244/326
A/Reunion/2245-3-M1E/2009(H1N1)	2009/08/17 (D0)	16	M	no	no	URTI	−/−/JQ431-343/205/290/273/245/327
A/Reunion/2336-4-M1E/2009(H1N1)	2009/08/11 (D0)	9	M	no	no	ILI	−/−/JQ431-340/197/301/274/231/328
A/Reunion/2378-1-M1E/2009(H1N1)	2009/08/24 (D0)	53	F	no	no	ILI	JQ431-379/366/338/221/307/255/249/329
A/Reunion/2433-3-M1E/2009(H1N1)	2009/08/21 (D0)	2	F	no	no	ILI	−/−/JQ431-341/196/302/256/232/330
A/Reunion/2692-2-M2E/2009(H1N1)	2009/09/02 (D3)	6	M	no	no	URTI	JQ431-388/367/363/220/280/275/246/331
A/Reunion/2923-3-M1E/2009(H1N1)	2009/08/20 (D0)	15	F	no	no	ILI	JQ431-382/373/336/213/303/276/247/332
A/Reunion/2956-1-M2E/2009(H1N1)	2009/09/04 (D3)	26	F	H5	no	ILI	−/−/JQ431-346/222/305/253/251/333
A/Reunion/2956-2-M1E/2009(H1N1)	2009/09/02 (D0)	8	M		no	ILI	−/−/JQ431-347/223/306/254/250/334
A/Reunion/2969-5-M1E/2009(H1N1)	2009/10/12 (D0)	3	M	no	no	ILI	JQ431-386/372/357/214/304/277/248/335

GenBank accession numbers of Reunion Island sequences determined in this study are presented.

* *Influenza*-like illness (ILI) is defined as documented fever (>37.8°C) associated with at least one Upper Respiratory Tract or systemic Infections (URTI). URTI is defined as at least two upper respiratory tract symptoms, such as myalgia, headache, aching, running nose, etc. with no documented fever. Neg is defined as no symptom or only one URTI symptom.

More phylogenetic analyses were conducted in detail for SWOI region, based on available sequences recovered from neighboring countries in the EPIFLU™ GISAID database and the previously selected 101 GenBank sequences. As sequences from EPIFLU™ GISAID database present only partial HA (1,174/1,710 nt) and NA (complete ORF) we limited our analysis to these portion of concatenation for all the used sequences.

The DNA substitution model that best fitted our data for both separate segments and concatemers was performed by the software jModelTest 0.1.1 [25] and was considered for phylogenetic and evolutionary analyses. We selected different models of nucleotide substitution using the corrected Akaike information criterion.

Phylogenetic trees were constructed by Maximum Likelihood (ML) within PHYML v.2.1b1 [26], according to the selected nucleotide substitution model. Nodal support was evaluated by 1000 bootstrap replicates. Bayesian phylogenetic Inference (BI) was carried out using MrBayes 3.1.2 [27]. BI for each data set based on the best-fitting model, was conducted with two independent runs of four incrementally heated, Metropolis Coupled Markov Chain Monte Carlo (MCMC) starting from a random tree. MCMC were run for 10,000,000 generations with trees and associated model parameters being sampled every 400 generations. The initial 2000 trees in each run were discarded as burning samples and the harmonic mean of the likelihood was calculated by combining the two independent runs.

### Estimation of Evolutionary Distance

Evolutionary distances between sequences of distinct phylogenetic groups in these analyses were calculated using MEGA 5 [28], taking into account the best model of sequences evolution allowing correction of the estimates of evolutionary distance. Since MEGA does not contain the HKY model proposed, we used the Tajima-Nei correction [29], which is the nearest model to those proposed by jModelTest.

### Molecular Clock Phylogenies and Date Estimation

Molecular clock phylogenies were estimated using the MCMC method and were calculated by using a maximum of available sequences from Reunion Island (Concat-6). GTR + Γ was used in the analysis as proposed by jModelTest. Only sequences for which full date information was available were retained for this estimation. The Bayesian MCMC analyses were performed using BEAST v.1.6.1 [30] under a strict molecular clock setting. An exponential-growth coalescent model was chosen as a prior on the tree. We used an UPGMA starting tree and ran a chain length of 50 million by sampling trees every 10,000 generations. Convergence and burning were assessed using Tracer v1.4.1b (http://tree.bio.ed.ac.uk/software/tracer/). The maximum clade credibility tree for analyzing the MCMC data set was annotated by TreeAnotator in the BEAST package. The tree was visualized using FigTree v1.2.2. (http://tree.bio.ed.ac.uk/software/figtree/).

### Mutation Analysis

Viral pH1N1 sequences isolated in 2009 from human hosts and for which full-genome information was available were downloaded from the EPIFLU™ GISAID database. Sequences containing residues specific to global clade 7 were identified (∼1,600 full-genome sequences) and concatenated. In order to identify mutations that were specific to the sequences from Reunion Island, by-eye comparison with global sequences was facilitated using Geneious Pro 5.3.4 [23].

### Ethics Statements

The protocol was conducted in accordance to the principles expressed in the Declaration of Helsinki and French law for biomedical research. This protocol was approved by the Ethical/Institution Review Board CPP (Comité de Protection des Personnes of Bordeaux 2 University) and as required by the French low regulation at AFSSAPS under the N°: ID RCB AFSSAPS: 2009-A00689-48. Every eligible person for participation was asked for giving their written informed consent. All participants gave their written informed consent. We also obtained informed written consent from the next of kin. All samples were de-identified and analyzed anonymously for the study.

## Results

The CoPanFlu-RUN study allowed collection of 1,196 nasal swabs belonging to 443 individuals and 125 households that were analysed for the presence of pH1N1. A total of 101 nasal swabs (8.4%) corresponding to 62 individuals (14.0%) tested pH1N1 positive. Amplified material from 28 different swabs (26 individuals from 21 households) was successfully characterised: 15 viruses were fully sequenced (8 segments, complete ORFs) and 13 were partially sequenced (6 segments: PA, HA, NP, NA, M and NS, complete ORFs). This includes 5 household-derived pairs of sequences (i.e. sequences obtained from two distinct individuals living in the same household), and 2 individual-derived pairs of sequences (i.e. sequences obtained from the same individual from two consecutive swabs [Table 1]). None of the individuals in our cohort population, including the 26 individuals from which the 28 sequences were obtained, developed severe clinical symptoms.

### Phylogenetic Analysis

In agreement with previous studies [31], [32], which have reported different mutation rates of the different segments of the *Influenza* viruses, jModelTest analysis demonstrated that different nucleotide substitution models were most relevant for the different genomic segments. ML analyses were carried out using the identified substitution model (GTR + Γ) for both Concat-6 and Concat-8. However, for the BI, we were able to apply the appropriate model to the analysis of each segment partition (HKY + Γ for HA, PA and PB1, GTR for NA, HKY for NS and M, HKY + I for NP and PB2).

#### Global context

**Figure 1:**
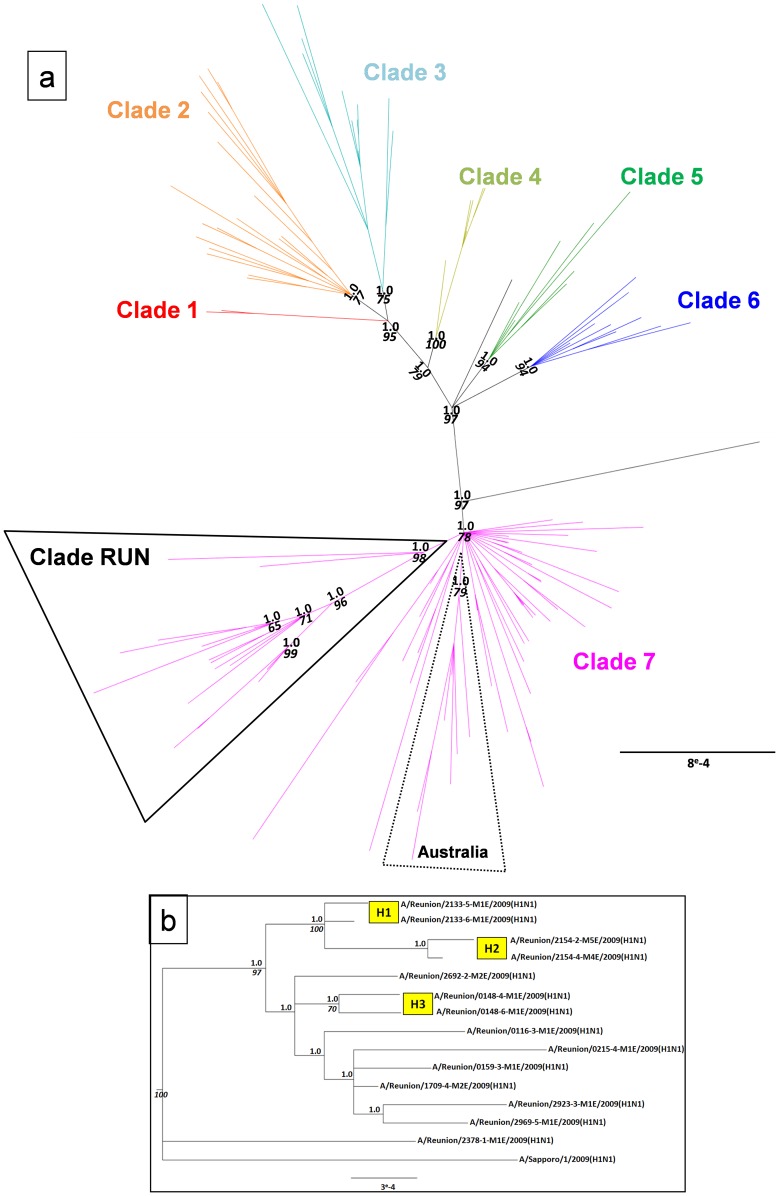
Full-genome pH1N1 phylogenetic analysis of Reunion Island viruses. **a.** Full-genome derived phylogenetic tree of concatenated sequences from 15 Reunion Island viruses and 101 representative sequences of the 7 world clades. Bayesian analyses were used to fix tree topologies. Branches are colored by global clade, as defined by Nelson et al. [5]. Phylogenetic clusters containing Reunion Island and Australian sequences are outlined. **b.** Enlarged representation of Clade RUN. “H” indicates viral sequences derived from the same Household. Throughout, Posterior Probabilities are represented in bold (PP>0.95), and where nodes coincided Maximum Likelihood bootstrap values are represented in italic (ML_bp_>70). Scale bar indicates the number of nucleotide substitution per site.

The global phylogenetic analyses conducted on concatemers of 8 (Figure 1) and 6 (Figure S1) segments from Reunion Island and GenBank sequences support the presence of 7 distinct global clades (major nodes have PP = 1.0 and BP_ML_ >71). Genetic distances between the 7 major clades varied from 0.14% to 0.22% (Table 2).

**Table 2:** Estimates of Evolutionary Divergence of pH1N1 among different clades.

CLADE	1	2	3	4	5	6	7	AUSTRALIA	RUN
**1**	–	*0,0003*	*0,0003*	*0,0003*	*0,0003*	*0,0003*	*0,0003*	*0,0003*	*0,0004*
**2**	0,0014	–	*0,0002*	*0,0002*	*0,0002*	*0,0002*	*0,0003*	*0,0003*	*0,0004*
**3**	0,0015	0,0017	–	*0,0003*	*0,0003*	*0,0003*	*0,0003*	*0,0004*	*0,0003*
**4**	0,0014	0,0016	0,0017	–	*0,0003*	*0,0003*	*0,0003*	*0,0003*	*0,0004*
**5**	0,0018	0,0019	0,0020	0,0014	–	*0,0002*	*0,0002*	*0,0003*	*0,0003*
**6**	0,0017	0,0019	0,0020	0,0014	0,0014	–	*0,0002*	*0,0003*	*0,0003*
**7**	0,0019	0,0021	0,0022	0,0016	0,0016	0,0016	–	*0,0001*	*0,0002*
**AUSTRALIA**	0,0023	0,0024	0,0025	0,0020	0,0020	0,0019	0,0014	–	*0,0002*
**RUN**	0,0025	0,0027	0,0028	0,0022	0,0022	0,0022	0,0016	0,0020	–

Genetic distances are displayed below the diagonal and standard error estimates, in italic, are shown above the diagonal. Analyses were conducted in MEGA5 [28] using the Tajima-Nei model [29].

Sequences from Reunion Island were found to cluster together and with a single sequence from Japan (A/Sapporo/1/2009(H1N1)) with strong nodal-support (PP = 1.0 and BP_ML_ = 93 for Concat-6; PP = 1.0 and BP_ML_ = 98 for Concat-8), generating a distinct phylogenetic group that was designated “clade RUN” (Figure 1). Genetic distances between clade RUN and other global clades were in the same range than those observed between each other global clades (Table 2). Clade RUN was most closely related to clade 7 (0.16%), and most distantly related to clade 3 (0.28%), suggesting that Clade RUN diverged from clade 7. A similar diversification was observed for some Australian sequences (7 of 8) that also formed a distinct group within clade 7 (PP = 1.0 and BP_ML_ = 85 for Concat-6; PP = 1.0 and BP_ML_ = 79 for Concat-8), with comparative genetic distances ranging from 0.14% (clade 7) to 0.25% (clade 3) (Table 2). Among the analysed set of sequences, no other strong geography-based clustering could be observed between the other members of clade 7, in agreement with previous studies [6], [33], [34].

Clade RUN sequences were then compared with 1597 pH1N1 clade 7 complete genome sequences retrieved from the EPIFLU™ GISAID database. When compared to the generated clade 7 consensus sequence, a total of 64 silent mutations, and 48 non-silent mutations were identified in all sequences obtained from the CoPanFlu-RUN cohort. Nucleotide changes that were common to more than one individual and that were uncommon or absent in all other included sequences are listed in Table S1.

Sequences belonging to clade 7 can be characterized by fixed amino acid changes in HA (S220T), NA (V106I) and NS1 (I123V) [5] (Table 3a). All sequences obtained from the CoPanFlu-RUN cohort contained each of these mutations. In addition, mutations NP (V100I) and NA (N248D), which are not systematically detected within clade 7 isolates were seen to be fixed in all sequences from clade RUN. These data confirm that clade RUN originated from clade 7. All clade RUN sequences also exhibited a fixed amino acid mutation in HA (D239E) and a silent mutation in NA (g873a). None of the Reunion sequences contained the specific mutations that are associated with oseltamivir resistance [35], [36].

**Table 3:** Specific clade mutations in discrete phylogenetic clades.

a								
	Gene							
Clade	PB2	PB1	PA	HA	NP	NA	M1–M2	NS1–NS2
**1**	–	–	**S 224 P**	**S 100 P**	–	–	–	–
				**A 214 T**				
				**V 338 I**				
**2**	–	–	**M 581 L**	–	T 373 I*	–	–	–
**3**	–	–	–	–	–	–	–	–
**4**	**V 649 I**	I 667 T*	–	–	V 100 I*	V 106 I*	–	E 63 K* (NS2)
**5**	–	–	–	–	**V 100 I**	**V 106 I**	–	–
						N 248 D*		
**6**	–	–	–	K 2 E*	V 100 I*	V 106 I*	–	–
				**Q 310 H**		N 248 D*		
**7**	–	–	–	S 220 T*	**V 100 I**	V 106 I*	–	I 123 V*
						**N 248 D**		
**RUN**	–	–	–	S 220 T*	V 100 I*	V 106 I*	–	I 123 V*
				D 239 E*		N 248 D*		
						g 873 a*		
**b**								
**RUN-A**	*g 120 a*	–	–	*c 42 a*	*a 366 g*	–	–	**N 133 D (NS1)**
	*g 1665 a*			*t 333 c*				
**RUN-B**	*a 807 g*	–	*c 1794 t*	–	–	*a 48 t*	*t 823 c*	–
	**V 414 I**							

Specific mutations within each clade are indicated. Fixed mutations, systematically present in all individuals of the indicated group, are indicated by*. Throughout, Silent DNA mutations are indicated in italic, amino acid mutations are highlighted in bold.

#### Local and community level context

In order to investigate genetic diversity within clade RUN, the maximum number of viral sequences from Reunion Island (n = 28) were considered in the phylogenetic analysis by considering the Concat-6 (Figure S1). Reunion Island sequences could be separated into two major sub-clades: sub-clade “RUN-A” clustering the majority of sequences (n = 25, PP = 1 and BP_ML_ = 94), while “RUN-B” forms a smaller sub-clade (n = 3, PP = 1 and BP_ML_ = 100) containing the sequence originating from Sapporo (Japan). RUN-A sequences were characterized by mutations in NS1 (N133D), NP (a366g), HA (c42a and t333c) and PB2 (g120a and g1665a), whereas RUN-B sequences lacked all these mutations (Table 3b). Fixed mutations were also observed for RUN-B sequences in PB2 (a807g and V414I), PA (c1794t), NA (a48t) and M (t823c). No correlation was found between the sub-clades and the temporal and geographical distributions of the individuals in Reunion Island, nor the clinical status of the individuals (data not shown).

Among the 28 viral sequences from Reunion Island, there were 5 household pairs of sequences (i.e. sequences generated from two different members of 5 households). Sequences derived from within households showed a high level of genetic similarity, with H2, H3 and H5 forming distinct evolutionary lineages with strong nodal support (Figures 1b and S1), suggestive of direct intra-household viral transmission. Similarly, the two individual pairs of sequences obtained from the same individual at d0 and d3 (I1 and I2) were also seen to cluster into distinct evolutionary lineages with strong nodal support, though genetic divergence was still observed between these sequences (0.01%–0.02%).

The mismatches between these related sequences (household members and individuals), are indicative of viral mutations occurring over a short period of time or relating to individual transmission events, and were identified for all segments (Table 4). The largest number of mismatches was observed in segments PB1 (5/14), PA (3/14) and M (3/14), whereas segments HA (1/14) and NA (0/14) showed little variation. Interestingly, each of the three mutations that were observed three days apart within the same individual was a reversion from a Reunion-specific sequence to that of the clade 7-consensus sequence, meaning that the sequences obtained at day 3 were likely more similar to the ancestral sequence than those obtained on the sample collected three days earlier. As the regeneration of a lost sequence by random mutation is extremely unlikely, this observation suggests the simultaneous presence of both reference and mutant viral quasispecies within these individuals with a shift in quasispecies dominance occurring over the course of three days. The presence of distinct quasispecies in these individuals was verified by analysis of the original sequence chromatograms, where regions with high coverage had differing but unambiguous chromatogram peaks at the corresponding positions (data not shown).

**Table 4:** Genetic sequence mismatches between related sequences.

	Segment								
	PB2	PB1	PA	NS	NP	NA	M	HA	Total
**I2**	–	–	0	1	0	0	1	0	**2**
**I1**	–	–	1	0	0	0	0	0	**1**
**H3**	0	2	1	0	0	0	2	1	**6**
**H4**	–	–	0	0	0	0	0	0	**0**
**H1**	0	2	1	0	0	0	0	0	**3**
**H2**	1	1	0	0	0	0	0	0	**2**
**H5**	–	–	0	0	0	0	0	0	**0**
**Total**	**1**	**5**	**3**	**1**	**0**	**0**	**3**	**1**	**14**

For each segment, the total number of DNA mismatches between individuals within a household (H) and sequences obtained from the same individual on separate days (I) is indicated (cf Table 1). “–” indicates where data was unavailable.

#### Context at the regional level

**Figure 2:**
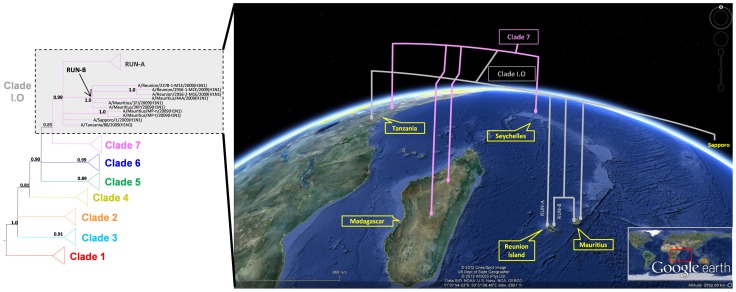
Illustration of the invasion of pH1N1 in the Indian Ocean (I.O) using an Euclidean drawing and a three-dimensional depiction. Phylogenetic tree derived from concatenated sequences of genomic segments HA (partial) and NA (complete ORF). Bayesian analyses were used to fix tree topologies. Branches are colored by global clade, as defined by Nelson et al. [5]. Posterior Probabilities are represented in bold (PP>0.80). Major clades were compressed in the classical (2D) tree for clarity. “Clade I.O” refers to all sequences of Clade RUN (n = 28) as well as Mauritian and Tanzanian sequences that were found to co-cluster (n = 6). Distinct strains of Seychellois, Malagasy and Tanzanian origin clustered within the compressed clade 7 (n = 8). 3D global representation, generated in Google Earth, depicts the phylogeny of all sequences derived from the Indian Ocean region. Satellite imagery: GoogleEarth. Date accessed: 19 04 2012. Co-ordinates: 11°37′54.03′′ 50°31′08.49′′E. Elevation: 2951 m. ©2012 Cnes/Spot Image.US Dept of State Geographer. ©2012 AfriGIS (Pty) Ltd. Data SIO, NOAA, US Navy, NGA, GEBCO.

In order to trace back early viral circulation of pH1N1 within the SWIO region, further phylogenetic analysis was performed using available sequences from viruses characterized in neighbouring countries. The analysis included segments for which sequence data was most commonly available (HA and NA). Viral strains from both Tanzania and Mauritius clustered with the sequences from the CoPanFlu-RUN-cohort suggesting that there had been an active circulation of this variant across the SWIO area. However, sequences from some SWIO islands (the Seychelles archipelago and Madagascar) did not cluster with those of clade RUN, suggesting circulation of multiple viral strains within the region at this time (Figure 2).

Mutations that had been identified as characteristic of clade RUN could be detected within some of the Mauritian and Tanzanian viral segments (Table 5). Available sequences of pH1N1 viruses isolated in Mauritius in August 2009 (at the peak of the epidemic in Reunion Island) could be ascribed to sub-clade RUN-B suggesting transmission of this *Influenza* virus between the two islands. Interestingly, although the sequences from the Tanzanian and Mauritian viruses identified in July, as well as those from the Japanese isolate from Sapporo in June, clustered with sequences from Reunion Island, these early regional sequences possessed none of the mutations which were associated with either of sub-clades RUN-A or RUN-B.

**Table 5:** Viral mutations of isolates from the South West Indian Ocean region.

Sequence name (from GISAID database)	Date	HA			NA	
		RUN	RUN-A		RUN	RUN-B
		*D 239 E*	*c 42 a*	*t 333 c*	*g 873 a*	*a 48 t*
A/Sapporo/1/2009	11/06/2009	yes	no	no	yes	no
A/Mauritius/MP-t/2009	01/07/2009	yes	no	no	yes	no
A/Mauritius/MP-n/2009	01/07/2009	yes	no	no	yes	no
A/Tanzania/88/2009	−/07/2009	yes	no	no	yes	no
A/Seychelles/106/2009	02/07/2009	no	no	no	no	no
A/Mauritius/1FJ/2009	05/08/2009	yes	no	no	yes	yes
A/Mauritius/4AA/2009	06/08/2009	yes	no	no	yes	yes
A/Mauritius/3KP/2009	06/08/2009	yes	no	no	yes	yes
A/Tanzania/381/2009	09/09/2009	no	no	no	no	no
A/Tanzania/386/2009	10/09/2009	no	no	no	no	no
A/Tanzania/393/2009	10/09/2009	no	no	no	no	no
A/Tanzania/424/2009	16/09/2009	no	no	no	no	no
A/Madagascar/9567/2009	04/11/2009	no	no	no	no	no
A/Madagascar/9551/2009	06/11/2009	no	no	no	no	no
A/Madagascar/10201/2009	17/12/2009	no	no	no	no	no

The presence of mutations with segments HA and NA that had been identified as specific to each genetic grouping (cf. Table 3) are indicated for isolates originating from the South West Indian Ocean region.

### Evolutionary Characteristics

**Figure 3:**
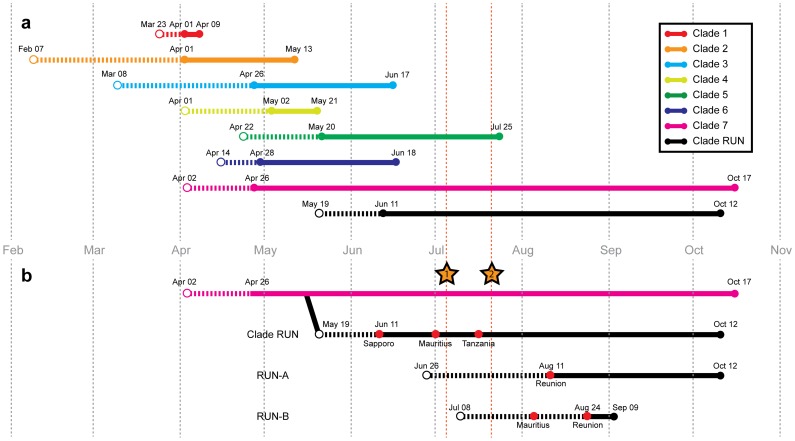
Date estimations of pH1N1 clade apparition and circulation. **a.** Bayesian estimates of The Most Recent Common Ancestor (TMRCA) for each clade (as shown in Figures 1 and S1). BEAST analysis is based on concatemers of 6 segments (complete ORF) of the 28 Reunion Island sequences and 101 globally-representative GenBank sequences. **b.** TMRCA of sub-clades RUN-A and RUN-B. Red points indicate strains originating from the South West Indian Ocean region that were identified as belonging to the specified sub-clades via identification of specific mutations in segments HA and NA (see Table 5). Throughout, points indicate date positions. Solid lines represent observed dates of an effective circulation for available isolates included in the analysis. Dashed lines represent estimated dates of circulation, dating back to the mean estimated TMRCA (95% confidence intervals). Lines are colored by clade as indicated. Stars n°1 and n°2 indicate the dates of the first imported case of pH1N1 (July 5) and the first autochthonous case (July 21) of pH1N1 in Reunion Island, respectively, as estimated by the regional epidemiological surveillance network.

The mean estimated dates of emergence for each clade are provided in figure 3. All of the obtained dates were in agreement with previously published studies [5], [6], [11], except for small differences in the estimated dates for clade 1 and 4 which can be explained by differences in sampling. Mean estimated dates of emergence were similar between Concat-8 and Concat-6 (data not shown).

The Most Recent Common Ancestor (TMRCA) of Clade RUN was estimated as May 19 and the estimated dates of emergence of sub-clades RUN-A and RUN-B were June 26 and July 8, respectively. TMRCA of Clade RUN suggests that this viral lineage was in circulation approximately 8–9 weeks before its initial detection in Reunion Island on July 5 and 11–12 weeks before the first autochthonous transmission (July 21) in Reunion Island detected by the epidemiological surveillance network. In addition, our previous observations of specific mutations in sequences obtained from Mauritius (strains MP-t and MP-n) and Tanzania (strain 88) show that regional circulation of pH1N1 preceded viral emergence in Reunion Island (Table 5).

## Discussion

Phylogenetic studies have demonstrated that pH1N1 virus has rapidly diversified into 7 discrete global clades. Even though clade 7 soon became largely prevailing while the other clades were gradually fading out in various geographic regions, studies in India [6], Japan [11], Argentina [8], Canada [14] and other countries have reported co-circulation of multiple pH1N1 strains belonging to different clades as a result of several viral introductions from different origins. In Reunion Island all amplified and sequenced pH1N1 viruses stem from clade 7, and were clustered in a local specific clade (clade RUN [Figures 1 & S1]). Evolutionarily, clade RUN was as distant from clade 7 as the other 6 global clades are from each other (Table 2). Viral sequences from Reunion Island have fixed all the mutations that had previously been identified as being specific to clade 7, including the [V100I]-NP and [N248D]-NA mutations which were not fixed among all clade 7 isolates [5]. In addition, clade RUN was characterized by 2 mutations that were systematically found in all local HA and NA sequences, respectively, the D239E mutation and the silent g873a mutation (Table 3).

There have been many descriptions of the HA D239E mutation [37], [38] including predictive structural studies [39]. It has been suggested that this residue likely plays an important structural role for the recognition and attachment of *Influenza* virus to its host receptor; changes at this position may therefore modulate the host immune response [38]. Recent studies in hospitalized patients suffering pH1N1 infection [40], [41] have shown that the D239G, D239N and D239E mutations were associated with severe (i.e. fatal cases reported), less severe, and mild illness, respectively [42]. Moreover, the D239E variant was found to preferentially colonize the upper respiratory tract, while D239G/N mutants also colonize the lower respiratory tract, hence causing severe acute respiratory syndromes, as is often observed in H5N1 *Influenza* [40]. Of note, none of the participants to the CoPanFlu-RUN cohort, infected by pH1N1, suffered serious medical complications. We have previously shown, based on serological data, that two thirds of individuals in Reunion Island that were infected by pH1N1 escaped medical detection by health services, likely because they developed only mild or inconspicuous disease [18]. This observation is in keeping with reports showing that infections with [D239E]-HA variants have generally speaking, been less severe than the one provoked by other variants.

Another salient observation from our population study is the clear division of clade RUN into two sub-clades, RUN-A and RUN-B. The divergence between RUN-A and RUN-B and their respective TMRCA estimation, suggest plausible multiple viral entries to Reunion Island even though they were closely related members of clade 7. Although the first report of pH1N1 infection detected in Reunion Island concerned a traveler coming from Australia [43], none of the selected Australian sequences appeared to share a common history with Reunion Island viruses. However, common history could be observed with available isolates from Tanzania and Mauritius (Figures 2, 3b and Table 5), with evidence of active circulation of multiple viral strains in the region but no solid conclusion could be made as to the origin of the genetic lineages present in Reunion Island.

Several reasons limit the extent of the conclusions that could be inferred from phylogenetic analyses of pH1N1: i) Despite the large number of pH1N1 genome sequences deposited in databases, the information is available only for a small subset of viruses that are circulating at the global level due to the rapid rate of *Influenza* mutation and spread; ii) Globalization means that autochthonous transmission results in complex patterns of viral emergence that are not limited by geographical boundaries. iii) A large number of sequenced *Influenza* isolates originate from patients suffering from severe *Influenza* illness; variants such as those from clade RUN that concerned individuals with mild or inconspicuous disease screened in the course of a prospective study conducted at the community level, represent a minority of sequences available in databanks. This point stresses the importance of prospective, community studies as an unbiased source of genetic material and a representation of the real natural history of disease in population.

Our estimates suggest that the common ancestral strain of Clade RUN emerged 8–9 weeks prior to the first identified case in Reunion Island and 11–12 weeks prior to the first case indicating autochthonous transmission (Figure 3). A similar conclusion was also drawn from Australian sequences [44]. A study reported from Taiwan [45] showed evidence of pre-epidemic subclinical community transmission as proved by seroconversion occurring several weeks before report of the first documented case in the island.

Our prospective study based on the follow-up of a community based cohort investigated viral transmission at the regional, community, intra-household levels as well as the temporal dynamics of *Influenza* viruses within an individual. Only few studies have developed so far a similar approach and they have focused only on the HA and HA/NA segments [46], [47]. These studies, as well as ours, highlight the fact that one can associate genetic variations to intra-household transmission of *Influenza* virus. However one should be aware that the variability observed in the incubation period, responsible for the “data stretch”, could lead to misinterpretations in transmission studies. Let us consider a household where two subjects 1 and 2 were concomitantly infected. Subject 1 has a shorter incubation period than subject 2, then subject 1 will be considered as an index case whereas subject 2 will be considered as a transmission case. Similarly, it could lead to a confusion of secondary and tertiary cases [48]. In such situations, mutation data are not sequential but rather linked to the original contaminating viral mixture.

Our experimental protocol did not include viral extraction from cell cultures in order to limit the risk of cell culture driven viral mutations. It has been reported by Zhirnov and collaborators that after isolation in cell culture (MDCK cell line) Influenza viruses often differ from those present in the clinical specimens, since adaptive changes occur during virus transmission from the human host to cells of heterologous origin [22]. Omitting the cell culture stage also offers the advantage of retaining the viral populations balance within the quasispecies mixture that was present in the first sample. As a consequence, we were able to observe in two individuals a rapid shift in the dominance of one viral population within the initial quasispecies. In each case, the characteristic mutation of the amplified material from the initial swab reverted, within 3 days, to the consensus sequence. Similar changes in quasispecies dominance were also observed between household members, and account for intra-household viral variability, reflecting the selection of viral population generated by the transmission bottleneck. The populations best fitted to the infected host, (hence, most represented within the quasispecies), are likely the ones that ultimately will emerge. Interestingly, studies have suggested that the genetic variation at the HA and NA levels, which are under the pressure of the host immune response, are mostly selected on the long-term [49]. Indeed, in our study the majority of shifts occurring on the short term, were not observed in segments HA and NA, but rather in PB1, PA and M, which are internal elements likely submitted to a different selective pressure. The quasispecies mixture that occurs within the individuals, as shown by deep sequencing studies [39], [41], [42], [50], [51], allows for diverse mutations to coexist over short periods of time during the pre-immune period. This phenomenon shows that the mechanisms to achieve best fit over the whole viral population at the individual host level, is at work following the inter-individual transmissions [52].

Most studies reported in the literature were conducted on single viral segments (generally HA or NA) of isolates from individuals with severe disease and hence have an inherently biased nature that can lead to important features of viral evolution being overlooked. Our observations highlight the importance of collecting data at the community level and conducting whole genome analysis to accurately understand viral dynamic.

## Supporting Information

Figure S1 **Partial genome pH1N1 phylogenetic analysis of Reunion Island viruses.** Phylogenetic tree derived from concatenated sequences of 6 genomic segments (Concat-6; PA, HA, NP, NA, M, NS) from 28 Reunion Island viruses and 101 representative sequences of the 7 world clades. Bayesian analyses were used to fix tree topologies. Branches are colored by global clade, as defined by Nelson et al. [5]. Posterior Probabilities are represented in bold (PP>0.95), Maximum Likelihood bootstrap values are represented in italic (ML_bp_>70). Scale bar indicates the number of nucleotide substitution per site. **Inset.** Enlarged representation of Clade RUN; “H” indicates viral sequences derived from the same Household, and “I” indicates viruses from the same individual in successive samples (three days apart). Arrows mark distinct phylogenetic clades RUN-A and RUN-B, indicated at major nodal junctions.
(TIF)

Table S1 **Characteristic mutations of pH1N1/2009** ***influenza*** **viral sequences from the CoPanFlu-RUN cohort.** Mutations were identified by comparison with reference sequences from closely-related viral strains from outside of Reunion Island. Only mutations that were present in more than one individual are included, and mutation prevalence within sequences obtained from the cohort is indicated. Characteristic mutations found in all sequences are highlighted in bold.
(DOC)
