# Supplementary figures and images for: Molecular Evolutionary Analysis of pH1N1 2009 Influenza Virus in Reunion Island, South West Indian Ocean Region: A Cohort Study
Source: PLoS One. 2012 Aug 27;7(8):e43742. doi: 10.1371/journal.pone.0043742 (PMC3428279; doi:10.1371/journal.pone.0043742)

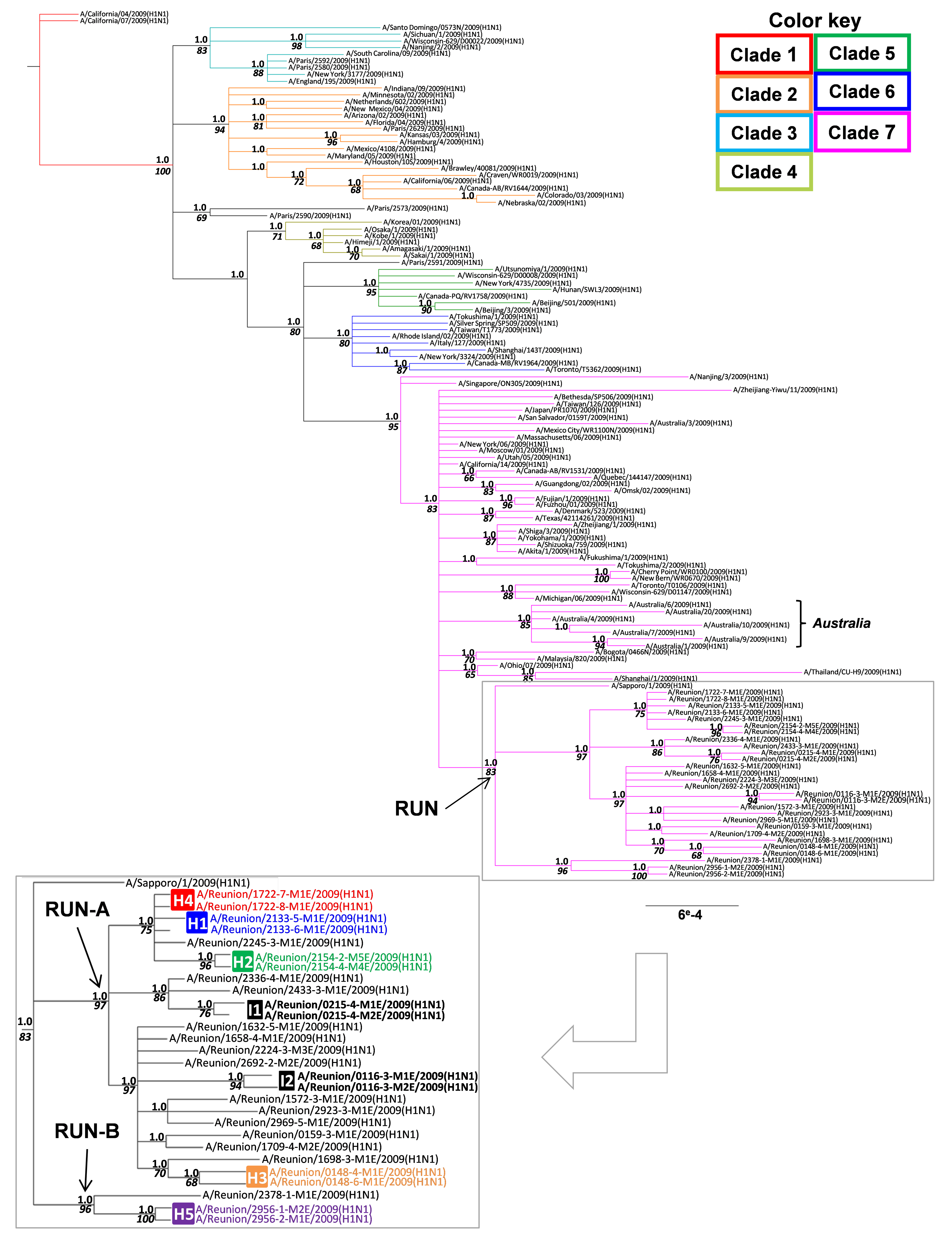

Supplement: Figure S1 — Partial genome pH1N1 phylogenetic analysis of Reunion Island viruses. Phylogenetic tree derived from concatenated sequences of 6 genomic segments (Concat-6; PA, HA, NP, NA, M, NS) from 28 Reunion Island viruses and 101 representative sequences of the 7 world clades. Bayesian analyses were used to fix tree topologies. Branches are colored by global clade, as defined by Nelson et al. [5]. Posterior Probabilities are represented in bold (PP>0.95), Maximum Likelihood bootstrap values are represented in italic (MLbp>70). Scale bar indicates the number of nucleotide substitution per site. Inset. Enlarged representation of Clade RUN; “H” indicates viral sequences derived from the same Household, and “I” indicates viruses from the same individual in successive samples (three days apart). Arrows mark distinct phylogenetic clades RUN-A and RUN-B, indicated at major nodal junctions. (TIF) [file pone.0043742.s001.tif]
