# Supplementary material for: Molecular Evolutionary Analysis of pH1N1 2009 Influenza Virus in Reunion Island, South West Indian Ocean Region: A Cohort Study
Source: PLoS One. 2012 Aug 27;7(8):e43742. doi: 10.1371/journal.pone.0043742 (PMC3428279; doi:10.1371/journal.pone.0043742)
Supplement: Table S1 — Characteristic mutations of pH1N1/2009 influenza viral sequences from the CoPanFlu-RUN cohort. Mutations were identified by comparison with reference sequences from closely-related viral strains from outside of Reunion Island. Only mutations that were present in more than one individual are included, and mutation prevalence within sequences obtained from the cohort is indicated. Characteristic mutations found in all sequences are highlighted in bold. (DOC) [file pone.0043742.s002.doc]

**Table S1. Characteristic mutations of pH1N1/2009 *influenza* viral sequences** from the CoPanFlu-RUN cohort.

| **Segment** | **Position (bp)** | **Codon** | **Consensus codon** | **Mutation type** | **Amino Acid** | **Number of individuals** |
| --- | --- | --- | --- | --- | --- | --- |
| PB2 | 120 | GAA | GAG | Silent | 40 E | 14/16 |
| PB2 | 807 | AGG | AGA | Silent | 269 R | 2/16 |
| PB2 | 1,240 | ATT | GTT | Non-Silent | V 414 I | 2/16 |
| PB2 | 1,452 | GGT | GGG | Silent | 484 G | 6/16 |
| PB2 | 1,665 | AGA | AGG | Silent | 555 R | 14/16 |
| PB1 | 470 | GAT | GCT | Non-Silent | A 157 D | 2/15 |
| PB1 | 634 | TTG | CTG | Silent | 212 L | 2/15 |
| PB1 | 1,419 | GTA | GTG | Silent | 473 V | 4/15 |
| PB1 | 2,039 | AAG | AGG | Non-Silent | R 680 A | 2/15 |
| PB1 | 2,112 | TCG | TCA | Silent | 704 S | 2/15 |
| PA | 1,041 | GAT | GAC | Silent | 347 D | 2/28 |
| PA | 1,135 | ATA | GTA | Non-Silent | V 379 I | 4/28 |
| PA | 1,330 | GAC | AAC | Non-Silent | N 444 D | 3/28 |
| PA | 1,794 | GCT | GCC | Silent | 598 A | 3/28 |
| PA | 2,022 | GAT | GAC | Silent | 674 D | 13/28 |
| PA | 2,129 | TCC | TTC | Non-Silent | F 710 S | 4/28 |
| NS | 28 | GAG | CAG | Non-Silent | Q 10 E (NS1) | 3/29 |
|  |  |  |  |  | Q 10 E (NS2) |  |
| NS | 397 | GAC | AAC | Non-Silent | N 133 D (NS1) | 26/29 |
| NP | 366 | CAG | CAA | Silent | 122 Q | 26/29 |
| NP | 576 | GAA | GAG | Silent | 192 E | 2/29 |
| NP | 735 | AGC | AGT | Silent | 245 S | 8/29 |
| NP | 1,119 | ACT | ACC | Silent | 373 T | 2/29 |
| NA | 48 | ACT | ACA | Silent | 16 T | 3/29 |
| NA | 603 | GGA | GGG | Silent | 201 G | 2/29 |
| **NA** | **873** | **GTA** | **GTG** | **Silent** | **291 V** | **29/29** |
| NA | 1,092 | AGT | AGC | Silent | 364 S | 2/29 |
| NA | 1,156 | CAC | AAC | Non-Silent | N 386 H | 2/29 |
| NA | 1,263 | TGT | TGC | Silent | 421 C | 2/29 |
| M | 207 | CCT | CCC | Silent | 69 P (MP1) | 5/29 |
| M | 543 | CTG | CTA | Silent | 181 L (MP1) | 2/29 |
| M | 816 | ATT | ACT | Non-Silent | T 43 I (MP2) | 4/29 |
| M | 823 | CGC | CGT | Silent | 45 R (MP2) | 3/29 |
| HA | 42 | ACA | ACC | Silent | 14 T | 26/29 |
| HA | 333 | GAC | GAT | Silent | 111 D | 26/29 |
| HA | 459 | TGC | TGT | Silent | 153 C | 8/29 |
| HA | 630 | CAA | CAG | Silent | 210 Q | 2/29 |
| HA | 634 | ACA | GCA | Non-Silent | A 212 T | 2/29 |
| HA | 687 | CCA | CCG | Silent | 229 P | 4/29 |
| **HA** | **717** | **GAA** | **GAT** | **Non-Silent** | **D 239 E** | **29/29** |
| HA | 1,012 | ATC | GTC | Non-Silent | V 338 I | 2/29 |
| HA | 1,506 | GAT | GAC | Silent | 502 D | 2/29 |
| HA | 1,564 | TTG | CTG | Silent | 522 L | 2/29 |

Mutations were identified by comparison with reference sequences from closely-related viral strains from outside of Reunion Island. Only mutations that were present in more than one individual are included, and mutation prevalence within sequences obtained from the cohort is indicated. Characteristic mutations found in all sequences are highlighted in bold.
